# Supplementary material for: Test–retest reliability of meta analytic networks during naturalistic viewing
Source: PLoS One. 2026 May 6;21(5):e0346967. doi: 10.1371/journal.pone.0346967 (PMC13148682; doi:10.1371/journal.pone.0346967)
Supplement: S1 Table — (DOCX) [file pone.0346967.s001.docx]

| Source of Variation | Sum of Squares | Degree of Freedom | F value | p value |
| --- | --- | --- | --- | --- |
| Sample | 0.005720221 | 1 | 1.552142676 | 0.213313893 |
| Condition | 0.004707598 | 4 | 0.319343621 | 0.865070948 |
| Session | 0.000745431 | 4 | 0.050566926 | 0.995203964 |
| Residual | 2.174368539 | 590 | Nan | Nan |

**Supplementary Table S1:** ANOVA revealed that there was no statistically significant difference in framewise displacement between the samples, conditions or sessions.
