# Supplementary material for: Test–retest reliability of meta analytic networks during naturalistic viewing
Source: PLoS One. 2026 May 6;21(5):e0346967. doi: 10.1371/journal.pone.0346967 (PMC13148682; doi:10.1371/journal.pone.0346967)
Supplement: S2 Table — (DOCX) [file pone.0346967.s002.docx]

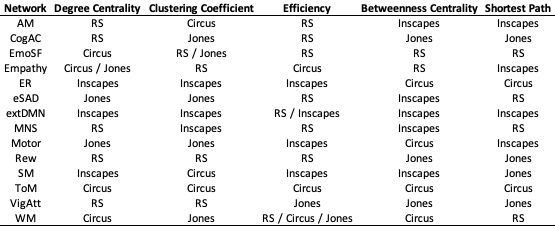


Supplementary Table 2: Condition yielding the highest ICC for each functional network and graph metric in IMAX.
