# Supplementary material for: Test–retest reliability of meta analytic networks during naturalistic viewing
Source: PLoS One. 2026 May 6;21(5):e0346967. doi: 10.1371/journal.pone.0346967 (PMC13148682; doi:10.1371/journal.pone.0346967)
Supplement: S4 Table — (DOCX) [file pone.0346967.s004.docx]

| network | node number | x | y | z | hemisphere | abbreviation |
| --- | --- | --- | --- | --- | --- | --- |
| AM | 1 | -1 | -53 | 21 | left | lPrc |
|  | 2 | -26 | -28 | -17 | left | lHippo |
|  | 3 | -49 | -61 | 31 | left | lTPJ |
|  | 4 | -2 | 51 | -11 | left | lFP |
|  | 5 | -60 | -9 | -18 | left | lSTS |
|  | 6 | -50 | 27 | -12 | left | lSOrbG |
|  | 7 | 26 | -33 | -15 | right | rHippo |
|  | 8 | -1 | 20 | 57 | left | lmSFG |
|  | 9 | 55 | -58 | 30 | right | rTPJ |
|  | 10 | -47 | 9 | 46 | left | lPrG |
|  | 11 | -42 | 53 | 7 | left | lFP |
|  | 12 | 26 | -14 | -23 | right | rHippo |
|  | 13 | 52 | -5 | -18 | right | rMTG |
|  | 14 | -39 | 13 | -41 | left | lTP |
|  | 15 | -38 | -82 | 38 | left | lIPL |
|  | 16 | -48 | 29 | 17 | left | lIFG |
|  | 17 | 52 | 31 | -11 | right | rSOrbG |
|  | 18 | -11 | 62 | 9 | left | lFP |
|  | 19 | 4 | -8 | 2 | right | rTh |
|  | 20 | -4 | 39 | 16 | left | lACC |
|  | 21 | -5 | -34 | 36 | left | lPCC |
|  | 22 | -29 | 16 | 51 | left | lSFG |
|  | 23 | 31 | 1 | -26 | right | rAm |
| CogAC | 1 | 36 | 22 | -4 | right | RaIns |
|  | 2 | 2 | 16 | 48 |  | preSMA |
|  | 3 | 48 | 12 | 30 | right | rIFGp |
|  | 4 | 36 | 2 | 54 | right | rdPMC |
|  | 5 | 48 | 30 | 24 | right | rIFGa |
|  | 6 | -38 | -44 | 46 | left | lIPS |
|  | 7 | -24 | -66 | 48 | left | lSPL |
|  | 8 | 40 | -46 | 46 | right | rIPS |
|  | 9 | 60 | -44 | 24 | right | rIPC |
|  | 10 | 30 | -62 | 52 | right | rSPL |
|  | 11 | -44 | 10 | 30 | left | lIFG |
|  | 12 | -34 | 20 | -4 | left | LaIns |
|  | 13 | -26 | 2 | 52 | left | ldPMC |
|  | 14 | 6 | -18 | -2 | right | rThal |
|  | 15 | -40 | -66 | -10 | left | lIOG |
|  | 16 | 48 | 19 | 6 | right | rIFG |
|  | 17 | 8 | 29 | 30 |  | aMCC |
|  | 18 | -45 | 27 | 30 | left | lIFG |
|  | 19 | 11 | 7 | 7 | right | rNcaud |
| eMDN | 1 | -46 | 6 | 30 | left | IFG_l |
|  | 2 | 50 | 12 | 28 | right | IFG_r |
|  | 3 | -32 | 20 | 2 | left | aIns_l |
|  | 4 | 36 | 22 | 0 | right | aIns_r |
|  | 5 | -4 | 14 | 44 | left | SMA_l |
|  | 6 | 6 | 18 | 46 | right | SMA_r |
|  | 7 | -32 | -52 | 46 | left | IPS_l |
|  | 8 | 32 | -58 | 48 | right | IPS_r |
|  | 9 | 44 | 36 | 20 | right | MFG_r |
|  | 10 | -28 | -4 | 52 | left | dPMC_l |
|  | 11 | -44 | 32 | 22 | left | MFG_l |
|  | 12 | 32 | 0 | 52 | right | dPMC_r |
|  | 13 | -20 | 6 | 4 | left | Put_l |
|  | 14 | 10 | -12 | 8 | right | Thal_r |
|  | 15 | -46 | -60 | -10 | left | ITG_l |
|  | 16 | 22 | 6 | 4 | right | Put_r |
|  | 17 | -10 | -16 | 6 | left | Thal_l |
| EmoSF | 1 | 4 | 47 | 7 |  | medPFC/pACC |
|  | 2 | 42 | 25 | 3 | right | rIFG |
|  | 3 | -42 | 25 | 3 | left | lIFG |
|  | 4 | 48 | 17 | 29 | right | rMFG/IFJ |
|  | 5 | -42 | 13 | 27 | left | lMFG/IFJ |
|  | 6 | -2 | 8 | 59 |  | SMedGy |
|  | 7 | 20 | -4 | -15 | right | rAm |
|  | 8 | -20 | -6 | -15 | left | lAm |
|  | 9 | -20 | -33 | -4 | left | lPHG |
|  | 10 | 14 | -33 | -7 | right | rPHG |
|  | 11 | 53 | -50 | 4 | right | rMTG |
|  | 12 | 38 | -55 | -20 | right | rFFG/FG3 |
|  | 13 | -40 | -55 | -22 | left | lFFG/FG3 |
|  | 14 | 38 | -76 | -16 | right | rpFFG/v4Cb |
|  | 15 | -40 | -78 | -21 | left | lpFFG/v4/Cb |
|  | 16 | -4 | 52 | 31 |  | medPFC |
|  | 17 | 36 | 25 | -3 | right | rOFC/Ins |
|  | 18 | -38 | 25 | -8 | left | lOFC/Ins |
|  | 19 | 2 | 19 | 25 | right | rACC/aMCC |
|  | 20 | 0 | -15 | 10 |  | Th |
|  | 21 | -2 | -31 | -7 |  | Pulv |
|  | 22 | -28 | -70 | -14 | left | lFFG/FG1 |
|  | 23 | 46 | -68 | -4 | right | rOcC/ITG |
|  | 24 | -48 | -72 | -4 | left | lOcC/ITG |
| Empathy | 1 | 2 | 56 | 18 |  | dmPFC |
|  | 2 | -8 | 54 | 34 |  | dmPFC |
|  | 3 | 36 | 22 | -8 | right | raI |
|  | 4 | -30 | 20 | 4 | left | laI |
|  | 5 | 50 | 12 | -8 | right | rIFG |
|  | 6 | 54 | 16 | 20 | right | rIFG/Area44 |
|  | 7 | 50 | 30 | 4 | right | rIFG/Area45 |
|  | 8 | -44 | 24 | -6 | left | lIFG |
|  | 9 | -4 | 18 | 50 |  | SMA |
|  | 10 | -2 | 28 | 20 |  | aMCC |
|  | 11 | -4 | 42 | 18 |  | rACC |
|  | 12 | -2 | -32 | 28 |  | PCC |
|  | 13 | 52 | -58 | 22 | right | rTPJ |
|  | 14 | -56 | -58 | 22 | left | lTPJ |
|  | 15 | 22 | -2 | -16 | right | rAm |
|  | 16 | 54 | -8 | -16 | right | rMTG |
|  | 17 | 52 | -36 | 2 | right | rpSTS |
|  | 18 | -12 | -4 | 12 | left | laTh |
|  | 19 | 6 | -32 | 2 | right | rpTh |
|  | 20 | 26 | -26 | -12 | right | rHippo |
|  | 21 | 2 | -20 | -12 |  | Midbrain |
|  | 22 | 14 | 4 | 0 | right | rGP |
| ER | 1 | 48 | 24 | 9 | right | rIFG |
|  | 2 | 42 | 21 | 45 | right | rMFG |
|  | 3 | 9 | 30 | 39 | right | RaMCC |
|  | 4 | 0 | -9 | 63 |  | SMA |
|  | 5 | -3 | 24 | 30 | left | LaMCC |
|  | 6 | -33 | 3 | 54 | left | lMFG |
|  | 7 | -36 | 21 | -3 | left | LaI |
|  | 8 | -42 | 45 | -6 | left | lIFG |
|  | 9 | 63 | -51 | 39 | right | rIPS |
|  | 10 | -42 | -66 | 42 | left | rSPL |
|  | 11 | -63 | -51 | -21 | left | lSTG |
|  | 12 | -51 | -39 | 3 | left | lMTG |
|  | 13 | 30 | -3 | -15 | right | rAmy |
|  | 14 | -18 | -3 | -15 | left | lAmy |
| eSAD | 1 | 0 | 38 | 10 |  | ACC |
|  | 2 | -24 | -10 | -20 | left | AmyHipp_L |
|  | 3 | 24 | -8 | -22 | right | AmyHipp_R |
|  | 4 | -2 | -52 | 26 |  | PrC |
|  | 5 | -2 | 32 | -8 |  | SGC |
|  | 6 | -46 | -66 | 18 | left | TPJ_L |
|  | 7 | 50 | -60 | 18 | right | TPJ_R |
|  | 8 | -2 | 52 | 14 |  | dmPFC |
|  | 9 | -6 | 10 | -8 | left | vBG_L |
|  | 10 | 6 | 10 | -8 | right | vBG_R |
|  | 11 | -2 | 50 | -10 |  | vmPFC |
|  | 12 | -54 | -10 | -20 | left | aMTS/aMTG |
| MNS | 1 | -56 | 8 | 28 | left | lPrG |
|  | 2 | -54 | 6 | 40 | left | lPrG |
|  | 3 | 58 | 16 | 10 | right | rIFG |
|  | 4 | 44 | -54 | -20 | right | rFP |
|  | 5 | -38 | -40 | 50 | left | lIPL |
|  | 6 | 51 | -36 | 50 | right | rIPL |
|  | 7 | -1 | 16 | 2 | left | lPMC |
|  | 8 | -54 | -50 | 10 | left | lPMTG |
|  | 9 | -52 | -70 | 6 | left | lOL |
|  | 10 | 54 | -64 | 4 | right | rOL |
|  | 11 | 30 | -62 | 63 | right | rSPL |
| Motor | 1 | -39 | -21 | 54 | left | lSMC* |
|  | 2 | 41 | -16 | 57 | right | rSMC* |
|  | 3 | -3 | -2 | 54 |  | SMA |
|  | 4 | -57 | 2 | 32 | left | lPMCv |
|  | 5 | -53 | -24 | 21 | left | lIPC |
|  | 6 | 45 | -38 | 48 | right | rIPC |
|  | 7 | -23 | -7 | 1 | left | lBG |
|  | 8 | 25 | -8 | 3 | right | rBG |
|  | 9 | -22 | -52 | 26 | left | lCba |
|  | 10 | 18 | -54 | -22 | right | rCba |
| Rew | 1 | 12 | 10 | -6 | right | rNAc |
|  | 2 | -10 | 8 | -4 | left | lPall |
|  | 3 | 36 | 20 | -6 | right | rIns |
|  | 4 | -32 | 20 | -4 | left | lIns |
|  | 5 | 0 | 24 | 40 | left | dmPFC |
|  | 6 | 0 | 54 | -8 | left | medOFC |
|  | 7 | 24 | -2 | -16 | right | rAm |
|  | 8 | 6 | -14 | 8 | right | rTh |
|  | 9 | -6 | -16 | 8 | left | lTh |
|  | 10 | 0 | 8 | 48 |  | SMA |
|  | 11 | 8 | -18 | -10 | right | rBrainStem |
|  | 12 | -6 | -18 | -10 | left | lBrainStem |
|  | 13 | 2 | 44 | 20 |  | ACC |
|  | 14 | -24 | 2 | 52 | left | lMFG |
|  | 15 | -38 | -4 | 6 | left | lIns(Id3) |
|  | 16 | 24 | 40 | -14 | right | rMidOFC(Fo3) |
|  | 17 | -16 | 42 | -14 | left | lMidOFC(Fo3) |
|  | 18 | 40 | 32 | 32 | right | raMFG |
|  | 19 | -28 | -56 | 48 | left | lIPL(IPS) |
|  | 20 | 28 | -58 | 50 | right | rAG |
|  | 21 | 0 | -32 | 32 | left | PCC |
|  | 22 | -36 | 50 | 10 | left | lFP |
|  | 23 | -46 | 42 | -4 | left | lLOFC |
|  | 24 | 30 | 4 | 50 | right | rpMFG |
|  | 25 | -22 | 30 | 48 | left | lSFG |
| ToM | 1 | 0 | 52 | -12 |  | vmPFC |
|  | 2 | 2 | 58 | 12 |  | FP |
|  | 3 | -8 | 56 | 30 |  | dmPFC |
|  | 4 | 2 | -56 | 30 |  | Prc |
|  | 5 | 56 | -50 | 18 | right | rTPJ |
|  | 6 | -48 | -56 | 24 | left | lTPJ |
|  | 7 | 54 | -2 | -20 | right | rTP |
|  | 8 | -54 | -2 | -24 | left | lTP |
|  | 9 | 52 | -18 | -12 | right | rMTG |
|  | 10 | -54 | -28 | -4 | left | lMTG |
|  | 11 | 50 | -34 | 0 | right | rpSTS |
|  | 12 | -58 | -44 | 4 | left | lpSTS |
|  | 13 | 54 | 28 | 6 | right | rIFG |
|  | 14 | -48 | 30 | -12 | left | lIFG |
|  | 15 | 48 | -72 | 8 | right | rV5 |
| VigAtt | 1 | -2 | 8 | 50 |  | aParacentralL |
|  | 2 | 8 | 32 | 46 | right | rmpSFG |
|  | 3 | 0 | 26 | 34 |  | dMCC |
|  | 4 | 50 | 8 | 32 | right | rIFJ |
|  | 5 | 40 | 22 | -4 | right | raI |
|  | 6 | 46 | 36 | 20 | right | rIFS |
|  | 7 | -40 | -12 | 60 | left | lPrecentralG |
|  | 8 | -46 | -68 | -6 | left | lIOG |
|  | 9 | -48 | 8 | 30 | left | lIFJ |
|  | 10 | 62 | -38 | 17 | right | rTPJ |
|  | 11 | 8 | -12 | 6 | right | rTh |
|  | 12 | 32 | -90 | 4 | right | rMOG |
|  | 13 | -42 | 12 | -2 | left | laI |
|  | 14 | -10 | -14 | 6 | left | lTh |
|  | 15 | 6 | -58 | -18 |  | Cb |
|  | 16 | 44 | -44 | 46 | right | rIPL |
| WM | 1 | -32 | 22 | -2 | left | aIns_l |
|  | 2 | -48 | 10 | 26 | left | IFG_l |
|  | 3 | -46 | 26 | 24 | left | lPFCc_l |
|  | 4 | -38 | 50 | 10 | left | lPFCr_l |
|  | 5 | 36 | 22 | -6 | right | aIns_r |
|  | 6 | 50 | 14 | 24 | right | IFG_r |
|  | 7 | 44 | 34 | 32 | right | lPFCc_r |
|  | 8 | 38 | 54 | 6 | right | lPFCr_l |
|  | 9 | 2 | 18 | 48 |  | pmFC |
|  | 10 | -28 | 0 | 56 | left | psFC |
|  | 11 | 30 | 2 | 56 | right | psFC |
|  | 12 | -42 | -42 | 46 | left | IPS_l |
|  | 13 | -34 | -52 | 48 | left | SPL_l |
|  | 14 | -24 | -66 | 54 | left | SPLp_l |
|  | 15 | 42 | -44 | 44 | right | IPSa_r |
|  | 16 | 32 | -58 | 48 | right | IPSp_r |
|  | 17 | 16 | -66 | 56 | right | SPLp_r |
|  | 18 | -12 | -12 | 12 | left | Thal_l |
|  | 19 | -16 | 2 | 14 | left | Ncaud_l |
|  | 20 | -16 | 0 | 2 | left | GP_l |
|  | 21 | 12 | -10 | 10 | right | Thal_r |
|  | 22 | -34 | -66 | -20 | left | Cb_FG_l |
|  | 23 | 32 | -64 | -18 | right | Cb_FG_r |
| SM | 1 | -46 | -69 | 28 | left | lIPL |
|  | 2 | -50 | -56 | 31 | left | lAG |
|  | 3 | -64 | -44 | -4 | left | lMTGp |
|  | 4 | -47 | -24 | -17 | left | lMTGm |
|  | 5 | -40 | -12 | -30 | left | lITGm |
|  | 6 | -8 | -57 | 17 | left | pCC |
|  | 7 | -20 | 36 | 44 | left | lSFG |
|  | 8 | -53 | 27 | -4 | left | LlOFGinf |
|  | 9 | 54 | -59 | 30 | right | rIPL |
|  | 10 | 43 | -72 | 31 | right | rmPC |
|  | 11 | -1 | 51 | -7 | left | lOFCmed |
|  | 12 | -5 | 56 | 24 | left | lSFGmed |
|  | 13 | -31 | -34 | -16 | left | lFG |
|  | 14 | -8 | 29 | -10 | left | lOFCmed/sCC |
|  | 15 | -46 | 25 | 23 | left | lIFG |
|  | 16 | 64 | -41 | -2 | right | rMTGp |
|  | 17 | -43 | -53 | 55 | left | lIPL |
|  | 18 | -1 | -18 | 40 | left | pMCC/PCC |
|  | 19 | -2 | -56 | 46 | left | lPCu |
|  | 20 | 51 | 20 | 26 | right | rIFG |
|  | 21 | 64 | -38 | 32 | right | rSMG |
|  | 22 | -23 | 26 | -16 | left | LlmOFCinf |
|  | 23 | -5 | -39 | 40 | left | lPCC |

**Supplementary Table 4: Centroid Coordinates of the nodes of the meta-analytically defined networks.**
