# Supplementary material for: Test–retest reliability of meta analytic networks during naturalistic viewing
Source: PLoS One. 2026 May 6;21(5):e0346967. doi: 10.1371/journal.pone.0346967 (PMC13148682; doi:10.1371/journal.pone.0346967)
Supplement: S1 Fig — ICCs are compared to corresponding null distribution with 5,000 randomizations. Vertical lines indicate the observed values in each condition. Blue lines indicate 95% CIs. The red vertical line indicates the observed difference. Only plots with significant results after FDR-BH correction are shown. (DOCX) [file pone.0346967.s005.docx]

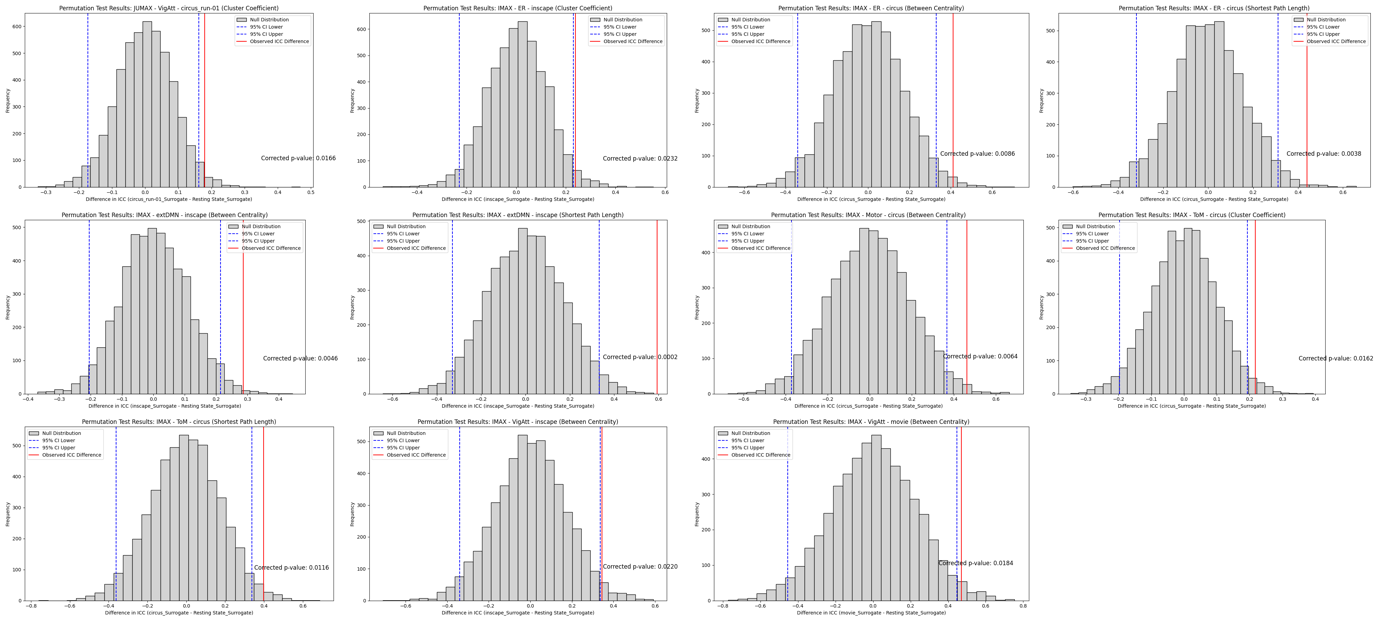


**Supplementary Figure S1:** Permutation tests of the reliability difference of different graph measures across RS and NV conditions. ICCs are compared to corresponding null distribution with 5,000 randomizations. Vertical lines indicate the observed values in each condition. Blue lines indicate 95% CIs. The red vertical line indicates the observed difference. Only plots with significant results after FDR-BH correction are shown.
