# Supplementary figures and images for: Test–retest reliability of meta analytic networks during naturalistic viewing
Source: PLoS One. 2026 May 6;21(5):e0346967. doi: 10.1371/journal.pone.0346967 (PMC13148682; doi:10.1371/journal.pone.0346967)

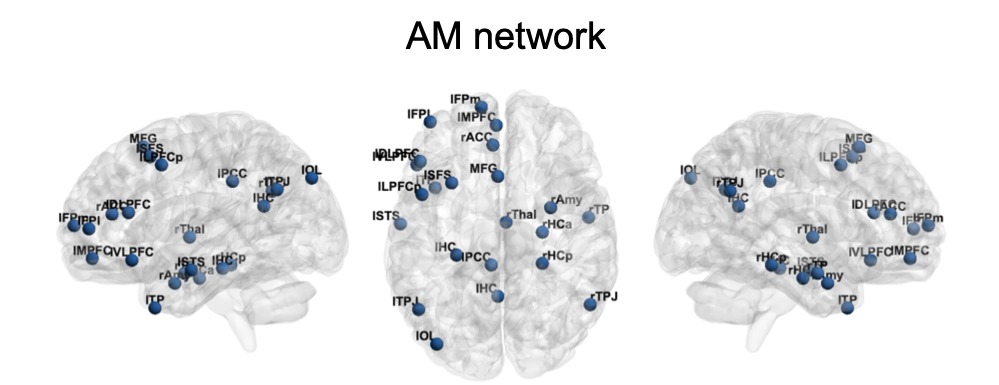

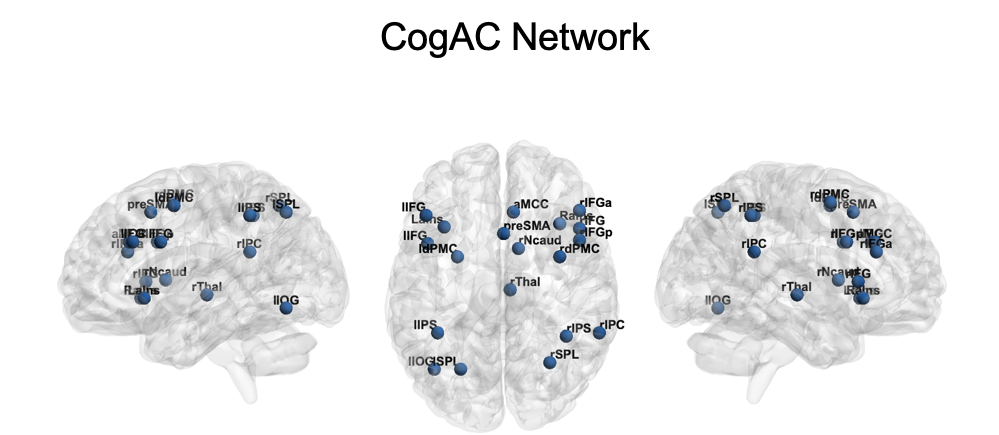

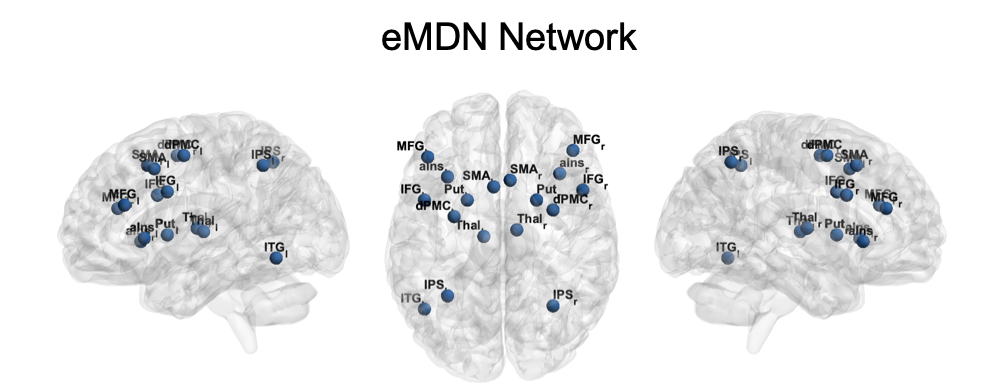

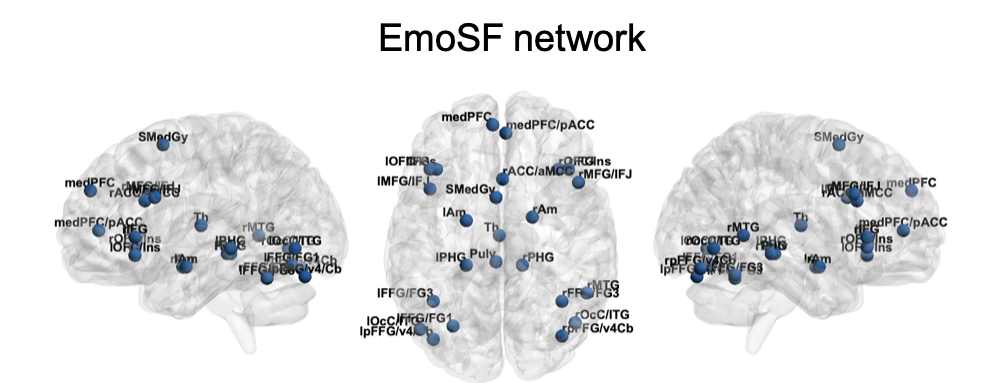

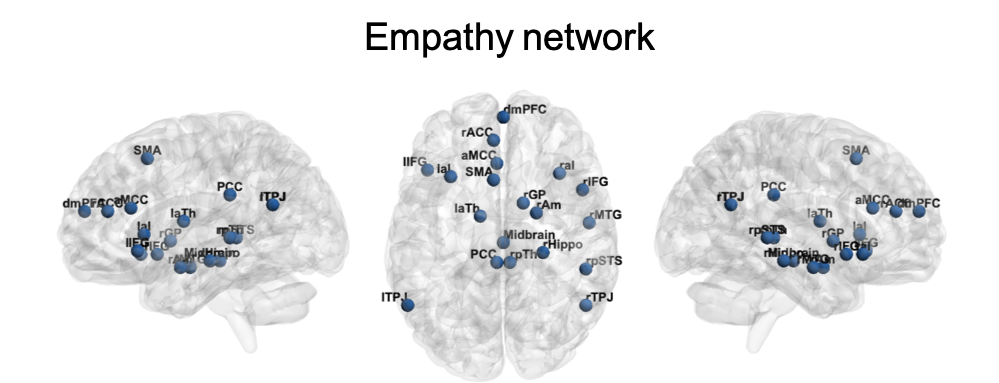

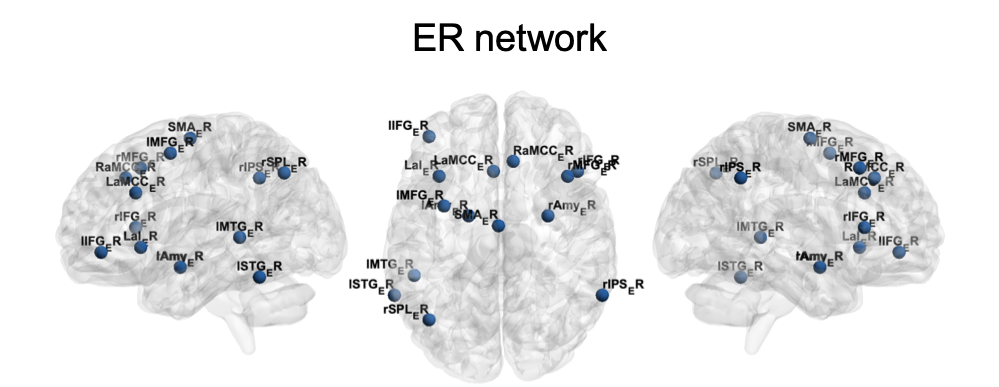

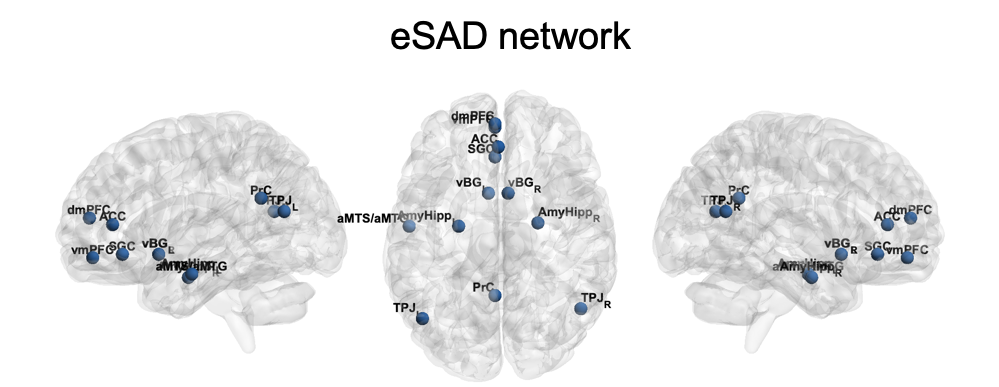

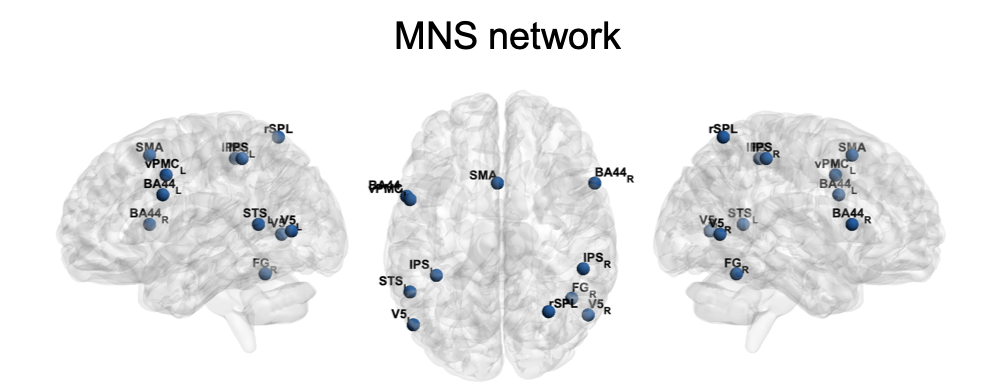

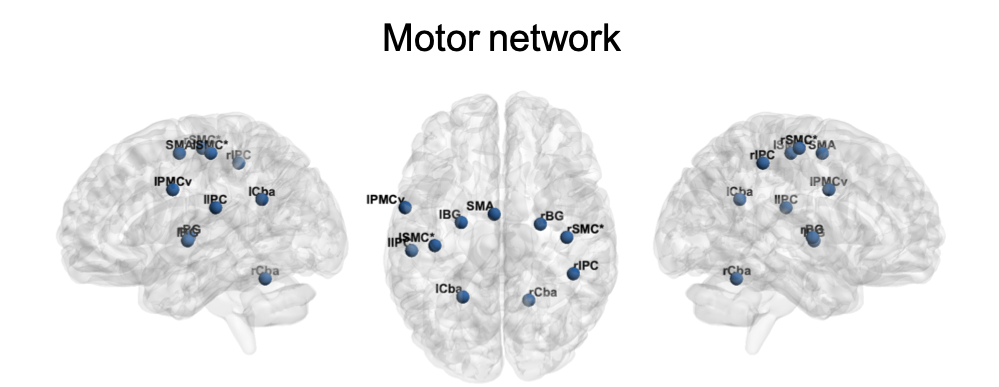

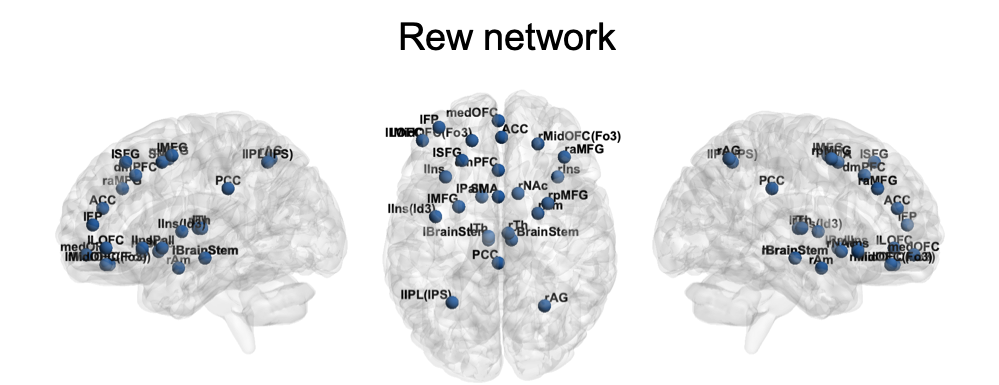

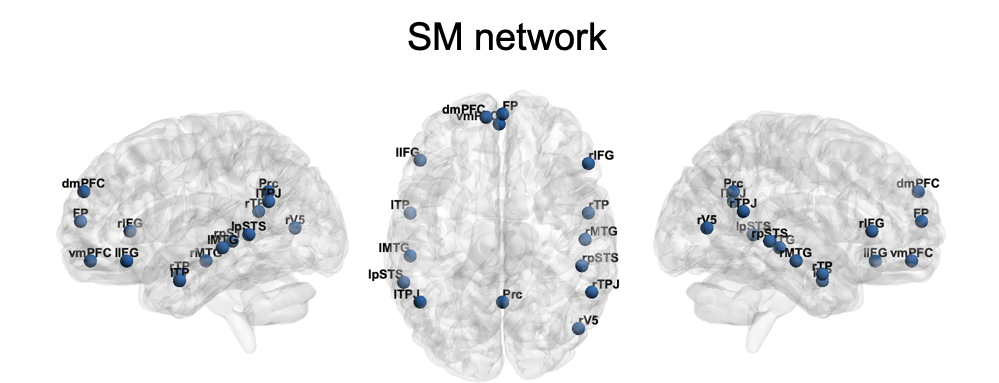


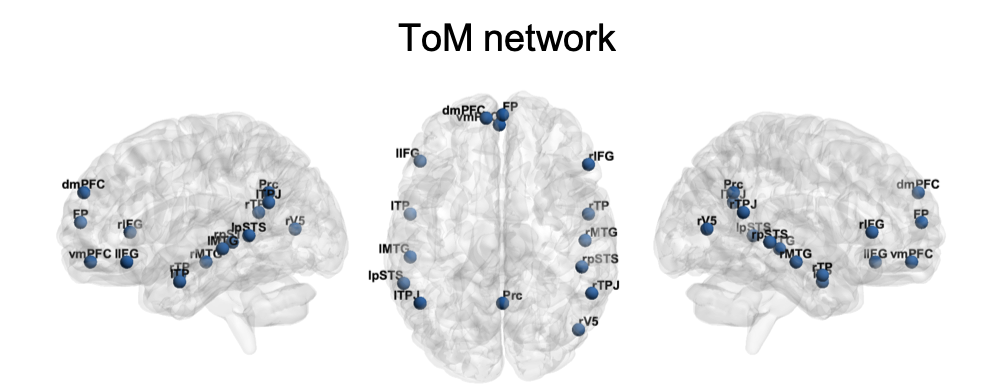


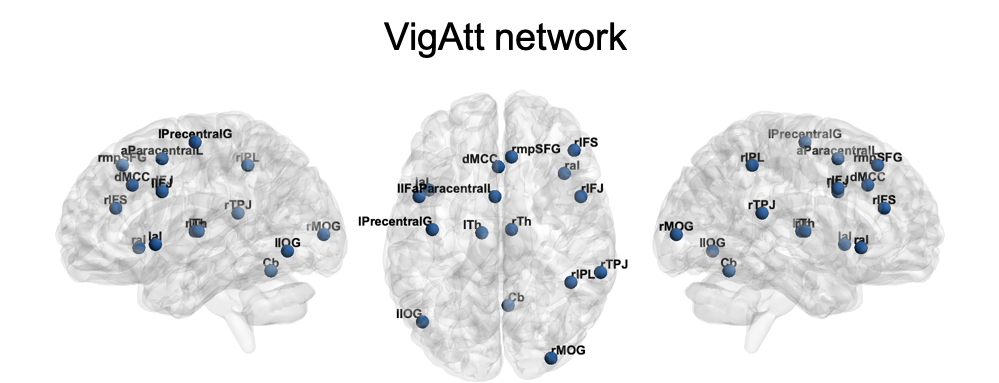

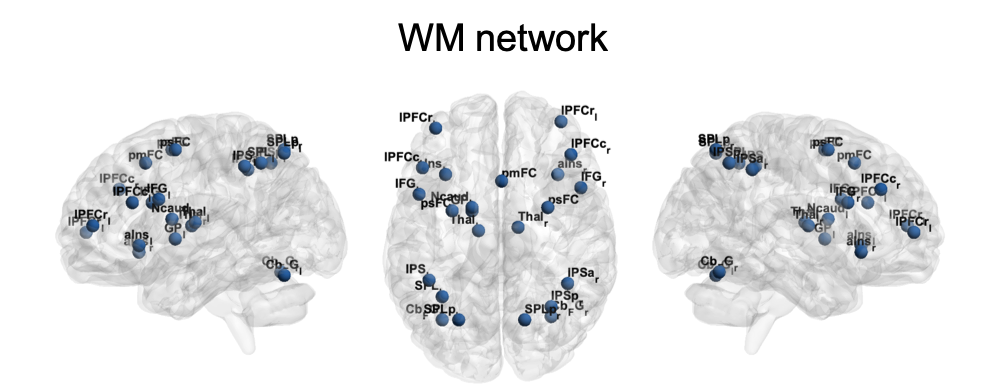


**Supplementary Figure S2: Node location of the meta-analytically defined networks**

Supplement: S2 Fig — (DOCX) [file pone.0346967.s006.docx]
